# Insulin resistance in cerebral small vessel disease: a mini review

**DOI:** 10.3389/fnins.2026.1760558

**Published:** 2026-02-03

**Authors:** Chen Su, Zhigang Cui, Junhong Guo

**Affiliations:** 1 First Clinical Medical College, Shanxi Medical University, Taiyuan, China; 2 Department of Neurology, First Hospital of Shanxi Medical University, Taiyuan, China; 3 Department of Neurology, The Third People's Hospital of Datong, Datong, Shanxi, China

**Keywords:** blood–brain barrier, cerebral small vessel disease, endothelial dysfunction, insulin resistance, treatment

## Abstract

Cerebral small vessel disease (CSVD) is a leading cause of stroke and vascular cognitive impairment, but its metabolic determinants are not fully understood. Emerging evidence indicates that insulin resistance (IR) plays a crucial role in CSVD through vascular, inflammatory, and oxidative mechanisms. Higher IR levels may be associated with greater burdens of white matter hyperintensities, lacunes, cerebral microbleeds, and enlarged perivascular spaces. Mechanistic studies suggest that IR impairs endothelial nitric oxide signaling, disrupts the blood–brain barrier, promotes vascular remodeling, and alters astrocytic aquaporin-4 polarization, which together aggravate both ischemic and hemorrhagic microvascular injury. Clinically, IR represents a modifiable target, and interventions that reduce IR, including the use of pioglitazone, metformin, glucagon-like peptide-1 receptor agonists, physical activity, and dietary modification, may help slow CSVD progression. This mini review summarizes current epidemiological and mechanistic evidence linking IR to CSVD and highlights the potential of metabolic regulation as a strategy to prevent or mitigate small-vessel–related brain injury.

## 1 Introduction

Cerebral small vessel disease (CSVD) encompasses a group of pathological processes affecting the small arteries, arterioles, venules, and capillaries of the brain (Wardlaw et al., 2019). The characteristic imaging features include white matter hyperintensities (WMH), lacunes, cerebral microbleeds (CMBs), enlarged perivascular spaces (EPVS), and brain atrophy (Wardlaw et al., 2013). CSVD is highly prevalent in the elderly population and has been recognized as a major contributor to stroke, cognitive decline, gait disturbances, and late-life depression (Ostergaard et al., 2016; Su et al., 2022; Castello et al., 2022). With the global demographic shift toward aging societies, the burden of CSVD is expected to increase substantially, making the identification of modifiable risk factors and the elucidation of underlying mechanisms of paramount importance.

Insulin resistance (IR), defined as a diminished sensitivity of peripheral tissues to insulin, represents a key metabolic abnormality that underlies type 2 diabetes and metabolic syndrome (Lee et al., 2022). Beyond its established role in cardiovascular disease, IR has recently gained attention as a potential contributor to cerebrovascular pathology (Tian et al., 2022; Frosch et al., 2017). While extensive research has focused on the link between IR and large-vessel atherosclerosis (Reardon et al., 2018), emerging evidence suggests that IR may also play a critical role in microvascular injury and, consequently, in the pathogenesis of CSVD (Nam et al., 2020; Teng et al., 2022). Notably, these effects may occur independently of overt diabetes, highlighting IR as an early and potentially modifiable risk factor (Wu et al., 2022).

Despite growing interest, the relationship between IR and CSVD remains insufficiently explored. Most existing studies have focused on diabetic populations or metabolic syndrome as a whole, while fewer have specifically addressed IR. Moreover, diverse surrogate indices have been used to quantify IR, which complicates comparisons across studies and limits clinical translation. This review aims to summarize current evidence linking IR with CSVD, discuss potential mechanisms, evaluate different IR indices, and highlight the clinical implications for prevention and management.

## 2 Assessment of IR

The hyperinsulinemic–euglycemic clamp is the gold standard for assessing IR but is impractical for large studies (Delai et al., 2022; Kim, 2009). Therefore, several surrogate indices have been developed using routine biochemical parameters. The homeostasis model assessment of insulin resistance (HOMA-IR) is the most commonly used, though it depends on fasting insulin assays (Anoop et al., 2021; Kosmas et al., 2024; Aliyu et al., 2025). It is calculated as fasting insulin (μU/mL) × fasting glucose (mmol/L) divided by 22.5. The triglyceride-glucose (TyG) index, calculated from fasting triglycerides and glucose, has gained popularity as a simple and reliable alternative that correlates well with clamp results and predicts metabolic and vascular outcomes (Son et al., 2022; Wu et al., 2021). It is defined as the natural logarithm of [fasting triglycerides (mg/dL) × fasting glucose (mg/dL)/2]. Other indices, including the metabolic score for insulin resistance (METS-IR) and the quantitative insulin sensitivity check index (QUICKI), also provide feasible measures of insulin sensitivity (Bello-Chavolla et al., 2018; Skrha et al., 2004). Among these, the TyG index appears particularly suitable for CSVD research because it is simple, reproducible, and applicable in large-scale epidemiological settings.

## 3 Relationship between IR and imaging findings of CSVD

### 3.1 White matter hyperintensities

WMH are hyperintense lesions on T2-weighted or fluid attenuated inversion recovery (FLAIR) magnetic resonance imaging (MRI), located in periventricular and deep white matter, and represent chronic ischemic injury of presumed vascular origin (Wardlaw et al., 2013). Multiple cross-sectional studies have demonstrated that IR is positively associated with WMH burden. In a Korean cohort of over 2,600 individuals, the TyG index showed a dose–response association with WMH volume, outperforming HOMA-IR in multivariable models (Nam et al., 2020). Similarly, in Japanese non-diabetic stroke patients, higher HOMA-IR independently predicted severe WMH grades (Katsumata et al., 2010). In the Maastricht Study, WMH volumes increased progressively from normoglycemia to prediabetes and type 2 diabetes, highlighting that metabolic dysfunction contributes to white matter injury even before overt diabetes develops (van Agtmaal et al., 2018). Nevertheless, prospective evidence is less consistent: in the Atherosclerosis Risk in Communities cohort, baseline IR score did not significantly predict 10-year WMH progression after full adjustment (Dearborn et al., 2015). The IR score was constructed via principal components analysis to capture combined effects of central obesity and insulin resistance, based on key variables like body mass index, waist measures, insulin, and HOMA-IR. Overall, a potential relationship between IR and WMH has been reported in cross-sectional studies, but longitudinal evidence remains limited or inconclusive.

### 3.2 Lacunes

Lacunes are defined as round or ovoid, cerebrospinal fluid-filled cavities (3–15 mm in diameter) in the deep gray or white matter, reflecting the chronic sequelae of small subcortical infarcts (Wardlaw et al., 2013). Both cross-sectional and longitudinal data support a robust association between IR and lacunes. In the cohort of non-diabetic adults, higher IR was significantly associated with lacunes, with mediation analyses showing that 30–40% of the effect was explained by elevated blood pressure (Zhou et al., 2024). In a large community-based cohort of nondiabetic adults in southeastern China, lower HOMA-IR was independently associated with a higher prevalence of lacunes and greater total CSVD burden (Zhou et al., 2022). Longitudinally, a cohort demonstrated that an IR composite score predicted incident lacunes over a decade, even when WMH progression was not significantly associated (Dearborn et al., 2015). Together, these findings suggest that IR may be an important risk factor for lacunes, potentially acting both directly and through its association with hypertension.

### 3.3 Cerebral microbleeds

CMBs appear as small hypointense lesions on T2-weighted or susceptibility-weighted MRI, reflecting hemosiderin deposits from prior microhemorrhage (Wardlaw et al., 2013). The evidence linking IR to CMBs is limited but emerging. A hospital-based study in older Chinese CSVD patients found that individuals in the highest HOMA-IR quartile had more than double the odds of CMBs compared with the lowest quartile, independent of age, blood pressure, and lipids (Li et al., 2023). In contrast, the Maastricht Study, conducted in a middle-aged community sample, found no significant association between prediabetes or diabetes and CMBs prevalence (van Agtmaal et al., 2018). These discrepancies may reflect differences in population risk profiles, lesion burden, and imaging sensitivity. Importantly, the distribution of CMBs reflects distinct pathologies. Deep CMBs, located in the basal ganglia, thalamus, or brainstem, are linked to hypertensive arteriopathy. Lobar CMBs, found in cortical and subcortical areas, are more typical of cerebral amyloid angiopathy (CAA) (Kuo et al., 2024). Current evidence suggests that IR may preferentially contribute to deep CMBs through mechanisms related to vascular remodeling and endothelial dysfunction in the context of metabolic syndrome (Hayden, 2024). However, most existing studies do not distinguish between deep and lobar lesions. Failure to account for this anatomical heterogeneity may obscure mechanistic links and dilute observed associations. Future longitudinal studies with topographic stratification of CMBs are needed to clarify whether IR specifically contributes to hypertensive-type microangiopathy versus amyloid-related vascular injury.

### 3.4 Enlarged perivascular spaces

EPVS are small, linear or ovoid fluid-filled structures along penetrating vessels, visible on T2-weighted MRI, and reflect impaired interstitial fluid clearance and microvascular dysfunction (Wardlaw et al., 2013). Emerging evidence implicates IR in EPVS burden. In a study of 235 non-diabetic Chinese older adults, higher HOMA-IR values were independently associated with a greater likelihood of moderate-to-severe basal ganglia EPVS, even after adjustment for conventional vascular risk factors (Wu et al., 2020). Another study using the TyG index showed significant associations with moderate-to-severe EPVS, particularly in the centrum semiovale (Cai et al., 2022). Although regional differences were noted, current studies suggest a potential link between IR and perivascular dysfunction. However, the evidence is limited, predominantly cross-sectional, and requires confirmation in longitudinal studies across diverse populations.

### 3.5 Global CSVD burden

Composite CSVD scores integrate WMH, lacunes, CMBs, and EPVS, offering a holistic measure of microvascular injury. Studies consistently report that insulin-resistant individuals have higher total CSVD scores. In a Chinese cohort of 156 non-diabetic adults, IR was significantly associated with greater CSVD burden, with a dose-dependent relationship between HOMA-IR levels and total CSVD score (Yang et al., 2019). In a larger cohort, IR was similarly linked to higher CSVD scores, with mediation analyses showing that 40–50% of the effect was explained by blood pressure (Zhou et al., 2024). These findings imply that IR may contribute to diffuse cerebral microvascular injury, potentially affecting multiple lesion types rather than a single marker.

Taken together, IR appears to be associated with multiple MRI features of CSVD, with the most consistent evidence for lacunes and EPVS. The link to CMBs and WMH is supported by preliminary studies but remains less conclusive (Table 1). Importantly, IR correlates with higher global CSVD burden, reinforcing its role as a systemic risk factor for diffuse microvascular injury. While part of this effect is mediated by hypertension and obesity, consistent independent associations suggest direct microvascular effects of IR. Longitudinal and interventional studies are needed to establish causality and to evaluate whether reducing IR can slow CSVD progression.

**Table 1:** Key studies examining the association between IR and CSVD imaging markers.

Study	Study design	Metabolic exposure	CSVD marker(s)	Main findings
Nam et al. (2020)	Cross-sectional	TyG index	WMH	Higher TyG index was associated with WMH volume
Katsumata et al. (2010)	Cross-sectional	HOMA-IR	WMH	Higher IR was associated with greater WMH severity
van Agtmaal et al. (2018)	Cross-sectional	OGTT	WMH, CMBs	Prediabetes and T2DM were associated with greater WMH burden, but not CMBs
Dearborn et al. (2015)	Longitudinal	IR score	WMH, Lacunes	IR score was associated with lacunes but not WMH progression
Zhou et al. (2024)	Cross-sectional	HOMA-IR	Lacunes, WMH, Global CSVD burden	Blood pressure partially mediated the association between IR and lacunes, WMH, and CSVD total burden
Zhou et al. (2022)	Cross-sectional	HOMA-IR, Insulin sensitivity index (ISI_0,120_)	Lacunes, WMH, Global CSVD burden	Higher IR was associated with greater lacunes, WMH, and CSVD total burden
Li et al. (2023)	Cross-sectional	HOMA-IR	CMBs	IR was associated with presence and burden of CMBs
Wu et al. (2020)	Cross-sectional	HOMA-IR	EPVS	Higher IR was independently associated with EPVS burden
Cai et al. (2022)	Cross-sectional	TyG index	EPVS	Elevated TyG index was associated with increased EPVS
Yang et al. (2019)	Cross-sectional	HOMA-IR	Global CSVD burden	IR was an independent risk factor for total CSVD burden

CMBs, cerebral microbleeds; CSVD, cerebral small vessel disease; EPVS, enlarged perivascular spaces; HOMA-IR, homeostasis model assessment of insulin resistance; IR, insulin resistance; TyG, triglyceride-glucose; OGTT, oral glucose tolerance test; WMH, white matter hyperintensities.

## 4 Pathophysiological mechanisms linking IR and CSVD

**Figure 1:**
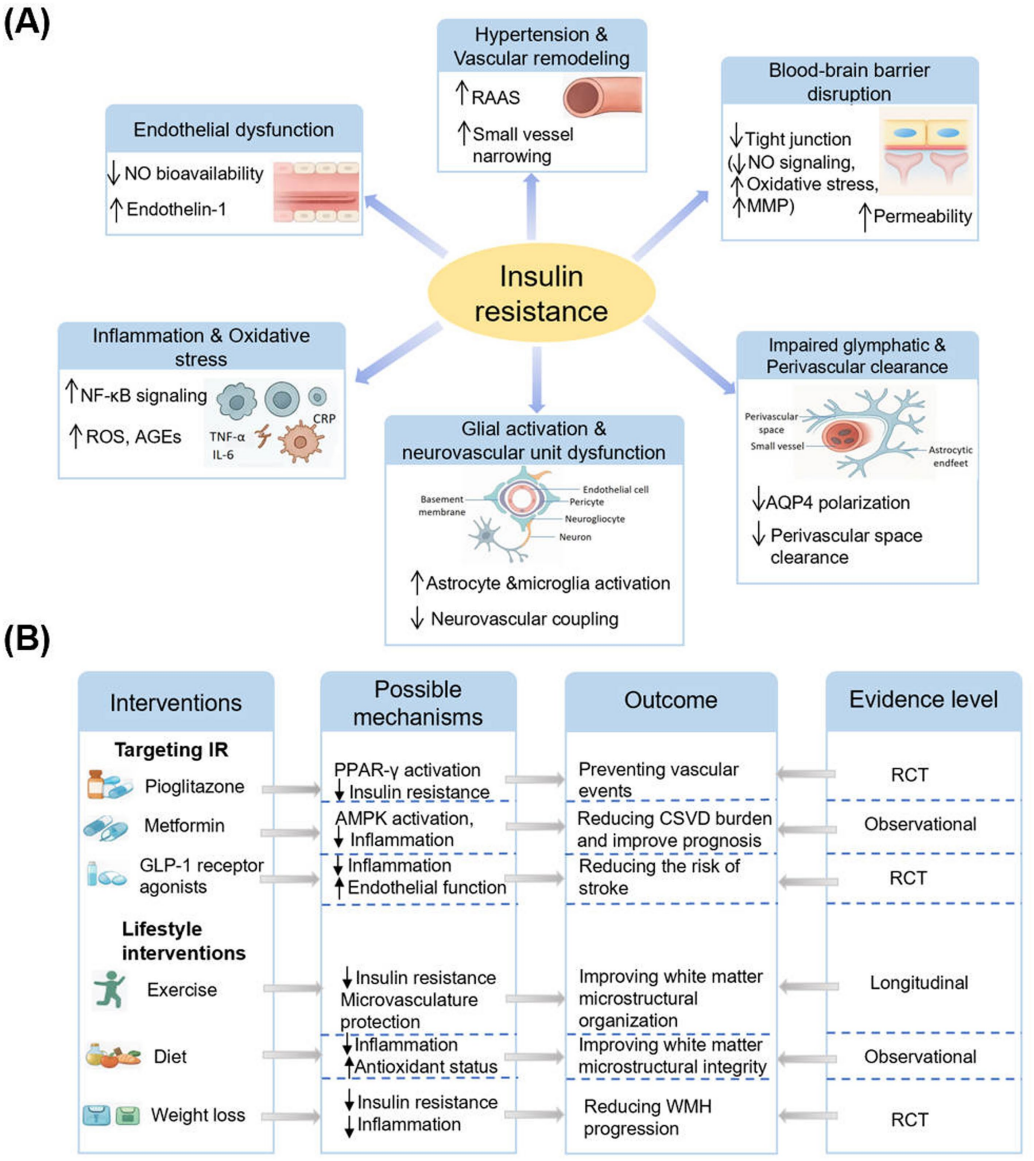
Mechanistic pathways linking insulin resistance to cerebral small vessel disease and potential interventions. **(A)** Insulin resistance may promote CSVD via endothelial dysfunction, BBB disruption, inflammation, vascular remodeling, impaired clearance, and glial activation. **(B)** Both pharmacologic and lifestyle interventions targeting insulin resistance show potential to mitigate CSVD-related brain injury through diverse mechanisms and evidence levels.

IR influences CSVD through a multifactorial network of interrelated mechanisms. These include endothelial dysfunction, blood–brain barrier (BBB) disruption, vascular remodeling via hypertension, chronic inflammation and oxidative stress, impaired glymphatic clearance, and neurovascular unit (NVU) dysfunction involving glial activation (Figure 1). Each of these processes contributes to the development of hallmark CSVD features such as WMH, lacunes, CMBs, and EPVS.

### 4.1 Endothelial dysfunction and impaired nitric oxide signaling

In healthy endothelium, insulin signaling activates the PI3K–Akt pathway, stimulating nitric oxide (NO) production and vasodilation (Hernandez-Resendiz et al., 2015). In IR, this pathway is selectively impaired, while the MAPK pathway remains active, leading to diminished NO bioavailability, increased endothelin-1 release, and heightened vasoconstriction (Quinones et al., 2005; Bai et al., 2022). Clinical studies link higher HOMA-IR or TyG index to reduced flow-mediated dilation and elevated circulating markers of endothelial injury (Zhu et al., 2024; Berezin et al., 2016). Experimental data further show that IR diminishes endothelial NO synthase (eNOS) activity, resulting in microvascular rarefaction and impaired cerebral autoregulation (Katakam et al., 2012; Carter et al., 2023). These abnormalities reduce perfusion in vulnerable white matter regions, predisposing to WMH and lacunes formation.

### 4.2 Blood–brain barrier disruption

The BBB maintains brain homeostasis through tight junctions between endothelial cells (Ashby and Mack, 2021). IR-related endothelial dysfunction destabilizes these junctions by reducing NO signaling, increasing oxidative stress, and upregulating matrix metalloproteinases (MMP)(Alshammari et al., 2024; Chen et al., 2025; de Aquino et al., 2018). Dynamic MRI studies demonstrate increased BBB permeability in IR and diabetic patients, even before overt CSVD lesions appear (Chen et al., 2021; Starr et al., 2003; Zhang et al., 2019). Disrupted barriers permit extravasation of plasma proteins and inflammatory mediators, which accumulate in perivascular spaces, exacerbate white matter injury, and promote CMBs formation (Jia et al., 2024). IR-related BBB impairment therefore contributes to both ischemic and hemorrhagic CSVD manifestations.

### 4.3 Hypertension and vascular remodeling

IR is closely linked to hypertension via sympathetic activation, sodium retention, and vascular stiffness (Sinha and Haque, 2022). Chronic hyperinsulinemia augments renin-angiotensin-aldosterone system (RAAS) activity and enhances vascular smooth muscle growth, promoting arteriolosclerosis and lipohyalinosis (Tanaka, 2020). Pathological remodeling narrows the lumen of penetrating arterioles, leading to chronic hypoperfusion and increased risk of vessel rupture (Katsi et al., 2024). Epidemiological mediation analyses confirm that blood pressure partially explains the association between IR and CSVD burden, particularly lacunes and WMH (Zhou et al., 2024). Thus, IR-driven hypertension acts as both a mediator and amplifier of microvascular pathology.

### 4.4 Inflammation and oxidative stress

IR represents a state of chronic low-grade inflammation characterized by elevated tumor necrosis factor-α (TNF-α), interleukin-6 (IL-6), and C-reactive protein (CRP)(Suren Garg et al., 2023). These mediators activate NF-κB signaling in endothelial cells, increasing adhesion molecule expression and leukocyte infiltration (Menon et al., 2023). Concurrently, oxidative stress from mitochondrial ROS and advanced glycation end-products (AGEs) damages vessel walls, uncouples eNOS, and promotes demyelination (Ji et al., 2022; Haase et al., 2024; Deng et al., 2018; He et al., 2019). Clinical data indicate that inflammatory biomarkers, including TNF-α, IL-6, and CRP, are positively correlated with CSVD lesion load (Zhang et al., 2022). Experimental studies further demonstrate that antioxidants ameliorate white matter damage in diabetic animal models (Wang et al., 2019; Infante-Garcia and Garcia-Alloza, 2019). Together, inflammation and oxidative stress provide a unifying mechanism for WMH progression, lacune formation, and microvascular fragility underlying CMBs (Wan et al., 2023).

### 4.5 Impaired glymphatic and perivascular clearance

The glymphatic system removes interstitial solutes via perivascular pathways (Iliff et al., 2012). IR impairs this system through multiple mechanisms. It causes arterial stiffening that reduces perivascular pulsatility, promotes vascular wall thickening that narrows perivascular channels, and induces astrocytic dysfunction that disturbs aquaporin-4 (AQP-4) polarization (Meng et al., 2023; Ozkan et al., 2023). Rodent models of diabetes demonstrate slowed interstitial clearance and cognitive decline (Deng et al., 2024), while human studies reveal associations between IR indices and increased EPVS burden (Wu et al., 2020; Cai et al., 2022). Impaired clearance leads to accumulation of metabolic wastes such as amyloid-β, further damaging vessel walls and aggravating CSVD pathology (Lee et al., 2024).

### 4.6 Glial activation and neurovascular unit dysfunction

The NVU coordinates neuronal activity with vascular responses. IR disrupts this system by inducing astrocytic gliosis, microglial activation, and pericyte degeneration (Hayden, 2019). Microglia in insulin-resistant states adopt a pro-inflammatory phenotype, releasing cytokines and proteases that injure myelin and endothelium (Sun and Mi, 2025; Jackson et al., 2020). Astrocytic dysfunction impairs gliovascular coupling, reducing adaptive vasodilation in response to neuronal demand (Masamoto et al., 2015). These changes result in chronic hypoperfusion, demyelination, and progressive WMH. Functional imaging studies corroborate impaired cerebrovascular reactivity in individuals with IR (Chantler et al., 2015), linking NVU dysfunction to cognitive decline in CSVD.

Taken together, IR exerts a multifaceted impact on the cerebral microvasculature. Endothelial dysfunction, BBB breakdown, hypertension, inflammation, impaired clearance, and glial activation converge to drive CSVD pathology. These mechanisms act synergistically, creating a self-perpetuating cycle of vascular injury. Future studies should focus on disentangling direct versus indirect effects of IR, clarifying longitudinal causal pathways, and evaluating whether interventions that reduce IR can prevent or mitigate CSVD progression.

## 5 Potential intervention strategies

Given the converging epidemiological and mechanistic evidence that IR contributes to the burden of CSVD, the clinical implications of these findings deserve attention. Recognition of IR as a modifiable metabolic abnormality provides opportunities for both risk stratification and therapeutic intervention in patients with CSVD (Figure 1).

Targeting IR pharmacologically represents a promising therapeutic approach. A large randomized controlled trial showed that pioglitazone reduced recurrent vascular events in non-diabetic patients with IR after ischemic stroke, supporting the therapeutic potential of targeting metabolic dysfunction in cerebrovascular disease (Kernan et al., 2016). Long-term metformin use has been linked to lower CSVD burden and better post-stroke outcomes in patients with CSVD, though causal effects remain to be validated in randomized trials (Teng et al., 2021; Akiyama et al., 2024). Glucagon-like peptide-1 receptor agonists, which improve metabolic control and reduce stroke incidence in large outcome trials, offer another promising class with potential cerebrovascular benefits (Stefanou et al., 2024). Although these agents have not yet been specifically validated for CSVD, their pleiotropic effects, including improvement of endothelial function, reduction of inflammation, and mitigation of vascular risk, make them attractive candidates for future investigations.

Lifestyle interventions remain the cornerstone of IR management and may indirectly protect the cerebral microvasculature. Aerobic exercise reduces IR, enhances cerebrovascular reactivity, and has been linked to healthier white matter integrity (Pani et al., 2022). Adherence to Mediterranean-style diets correlates with lower white matter lesion volumes and improved microstructural connectivity, likely via anti-inflammatory and antioxidative effects (Samuelsson et al., 2023). Sustained weight loss reduces IR and may modestly attenuate lesion progression (Espeland et al., 2016). Multidomain approaches, as tested in the Finnish Geriatric Intervention Study, integrate diet, exercise, and vascular risk management, and have shown cognitive benefits in at-risk populations, suggesting potential applicability to CSVD prevention (Ngandu et al., 2015).

## 6 Limitations and interpretation of current evidence

Despite accumulating evidence linking IR to CSVD, several important limitations should be considered when interpreting current findings. First, CSVD represents a heterogeneous spectrum of imaging phenotypes, including WMH, lacunes, CMBs, and EPVS, which may reflect partially distinct underlying pathophysiological processes. Associations observed for one CSVD marker may not necessarily generalize to others, and pooling these phenotypes may obscure marker-specific relationships.

Second, most available studies are cross-sectional in design, limiting causal inference. Although longitudinal data suggest that IR may precede progression of certain CSVD features, these findings remain inconsistent and are vulnerable to residual confounding. In particular, hypertension and obesity frequently coexist with IR and may act as important confounders or mediators, especially for deep perforator-related lesions. Disentangling the independent contribution of IR from these closely related vascular risk factors remains challenging.

Third, interventional evidence linking improvement of IR to changes in CSVD imaging outcomes is scarce. While pharmacological and lifestyle interventions targeting IR have demonstrated benefits on vascular events and cognitive outcomes, dedicated trials incorporating CSVD imaging endpoints are largely lacking. As a result, current conclusions regarding therapeutic modulation of CSVD through IR should be viewed as hypothesis-generating rather than definitive.

Overall, these limitations underscore the need for well-designed longitudinal studies and interventional trials with standardized CSVD imaging outcomes to clarify causal pathways and clinical relevance.

## 7 Conclusion and outlook

IR is an emerging and potentially modifiable contributor to CSVD. Based on current evidence, the following points summarize key insights:

While most evidence linking IR to CSVD is cross-sectional, emerging studies suggest associations across multiple imaging markers.

IR may influence CSVD through endothelial dysfunction, inflammation, and impaired vascular homeostasis.

Its modifiable nature makes IR a potential target for interventions aiming to preserve brain health.

Longitudinal and interventional studies with neuroimaging endpoints are needed to clarify causal pathways and therapeutic value.

## References

[ref1] Akiyama N. Yamashiro T. Ninomiya I. Uemura M. Hattori Y. Ihara M. . (2024). Neuroprotective effects of oral metformin before stroke on cerebral small-vessel disease. J. Neurol. Sci. 456:122812. doi: 10.1016/j.jns.2023.122812, 38043334

[ref2] Aliyu U. Toor S. M. Abdalhakam I. Elrayess M. A. Abou Samra A. B. Albagha O. M. E. (2025). Evaluating indices of insulin resistance and estimating the prevalence of insulin resistance in a large biobank cohort. Front Endocrinol (Lausanne) 16:1591677. doi: 10.3389/fendo.2025.1591677, 40421243 PMC12104043

[ref3] Alshammari M. A. Alshehri A. O. Alqahtani F. Khan M. R. Bakhrebah M. A. Alasmari F. . (2024). Increased permeability of the blood-brain barrier in a diabetic mouse model (Lepr(db)(/db) mice). Int. J. Mol. Sci. 25:7768. doi: 10.3390/ijms25147768, 39063010 PMC11276738

[ref4] Anoop S. S. Dasgupta R. Rebekah G. Jose A. Inbakumari M. P. Finney G. . (2021). Lipid accumulation product (LAP) as a potential index to predict risk of insulin resistance in young, non-obese Asian Indian males from southern India: observations from hyperinsulinemic-euglycemic clamp studies. BMJ Open Diabetes Res. Care 9:e002414. doi: 10.1136/bmjdrc-2021-002414, 34531243 PMC8449941

[ref5] Ashby J. W. Mack J. J. (2021). Endothelial control of cerebral blood flow. Am. J. Pathol. 191, 1906–1916. doi: 10.1016/j.ajpath.2021.02.023, 33713686

[ref6] Bai T. Yu S. Feng J. (2022). Advances in the role of endothelial cells in cerebral small vessel disease. Front. Neurol. 13:861714. doi: 10.3389/fneur.2022.861714, 35481273 PMC9035937

[ref7] Bello-Chavolla O. Y. Almeda-Valdes P. Gomez-Velasco D. Viveros-Ruiz T. Cruz-Bautista I. Romo-Romo A. . (2018). METS-IR, a novel score to evaluate insulin sensitivity, is predictive of visceral adiposity and incident type 2 diabetes. Eur. J. Endocrinol. 178, 533–544. doi: 10.1530/EJE-17-0883, 29535168

[ref8] Berezin A. E. Kremzer A. A. Cammarota G. Zulli A. Petrovic D. Martell-Claros N. . (2016). Circulating endothelial-derived apoptotic microparticles and insulin resistance in non-diabetic patients with chronic heart failure. Clin. Chem. Lab. Med. 54, 1259–1267. doi: 10.1515/cclm-2015-0605 26656612

[ref9] Cai Y. Chen B. Zeng X. Xie M. Wei X. Cai J. (2022). The triglyceride glucose index is a risk factor for enlarged perivascular space. Front. Neurol. 13:782286. doi: 10.3389/fneur.2022.782286, 35185759 PMC8854364

[ref10] Carter K. J. Ward A. T. Kellawan J. M. Harrell J. W. Peltonen G. L. Roberts G. S. . (2023). Reduced basal macrovascular and microvascular cerebral blood flow in young adults with metabolic syndrome: potential mechanisms. J. Appl. Physiol. (1985) 135, 94–108. doi: 10.1152/japplphysiol.00688.2022, 37199780 PMC10292973

[ref11] Castello J. P. Pasi M. Kubiszewski P. Abramson J. R. Charidimou A. Kourkoulis C. . (2022). Cerebral small vessel disease and depression among intracerebral hemorrhage survivors. Stroke 53, 523–531. doi: 10.1161/STROKEAHA.121.035488, 34587793 PMC8792169

[ref12] Chantler P. D. Shrader C. D. Tabone L. E. d'Audiffret A. C. Huseynova K. Brooks S. D. . (2015). Cerebral cortical microvascular rarefaction in metabolic syndrome is dependent on insulin resistance and loss of nitric oxide bioavailability. Microcirculation 22, 435–445. doi: 10.1111/micc.12209, 26014499 PMC4551443

[ref13] Chen A. Duan Y. Zhou S. Du F. Peng H. Zeng D. . (2025). Mesenchymal stem cells restore endothelial integrity and alleviate emotional impairments in a diabetic mouse model via inhibition of MMP-9 activity. Int. J. Mol. Sci. 26:3355. doi: 10.3390/ijms26073355, 40244194 PMC11989596

[ref14] Chen Y. C. Lu B. Z. Shu Y. C. Sun Y. T. (2021). Spatiotemporal dynamics of cerebral vascular permeability in type 2 diabetes-related cerebral Microangiopathy. Front Endocrinol (Lausanne) 12:805637. doi: 10.3389/fendo.2021.805637, 35087478 PMC8786705

[ref15] de Aquino C. C. Leitao R. A. Oliveira Alves L. A. Coelho-Santos V. Guerrant R. L. Ribeiro C. F. . (2018). Effect of hypoproteic and high-fat diets on hippocampal blood-brain barrier permeability and oxidative stress. Front. Nutr. 5:131. doi: 10.3389/fnut.2018.00131, 30687711 PMC6333637

[ref16] Dearborn J. L. Schneider A. L. Sharrett A. R. Mosley T. H. Bezerra D. C. Knopman D. S. . (2015). Obesity, insulin resistance, and incident small vessel disease on magnetic resonance imaging: atherosclerosis risk in communities study. Stroke 46, 3131–3136. doi: 10.1161/STROKEAHA.115.010060, 26451022 PMC4624467

[ref17] Delai A. Gomes P. M. Foss-Freitas M. C. Elias J. Antonini S. R. Castro M. . (2022). Hyperinsulinemic-Euglycemic clamp strengthens the insulin resistance in nonclassical congenital adrenal hyperplasia. J. Clin. Endocrinol. Metab. 107, e1106–e1116. doi: 10.1210/clinem/dgab767, 34693966

[ref18] Deng S. Hu Y. Chen S. Xue Y. Yao D. Sun Q. . (2024). Chronic sleep fragmentation impairs brain interstitial clearance in young wildtype mice. J. Cereb. Blood Flow Metab. 44, 1515–1531. doi: 10.1177/0271678X241230188, 38639025 PMC11418708

[ref19] Deng X. Huang W. Peng J. Zhu T. T. Sun X. L. Zhou X. Y. . (2018). Irisin alleviates advanced glycation end products-induced inflammation and endothelial dysfunction via inhibiting ROS-NLRP3 Inflammasome signaling. Inflammation 41, 260–275. doi: 10.1007/s10753-017-0685-3, 29098483

[ref20] Espeland M. A. Erickson K. Neiberg R. H. Jakicic J. M. Wadden T. A. Wing R. R. . (2016). Brain and white matter Hyperintensity volumes after 10 years of random assignment to lifestyle intervention. Diabetes Care 39, 764–771. doi: 10.2337/dc15-2230, 27208378 PMC4839171

[ref21] Frosch O. H. Yau P. L. Osorio R. S. Rusinek H. Storey P. Convit A. (2017). Insulin resistance among obese middle-aged is associated with decreased cerebrovascular reactivity. Neurology 89, 249–255. doi: 10.1212/WNL.0000000000004110, 28615420 PMC5513815

[ref22] Haase S. Kuhbandner K. Muhleck F. Gisevius B. Freudenstein D. Hirschberg S. . (2024). Dietary galactose exacerbates autoimmune neuroinflammation via advanced glycation end product-mediated neurodegeneration. Front. Immunol. 15:1367819. doi: 10.3389/fimmu.2024.1367819, 39185426 PMC11341352

[ref23] Hayden M. R. (2019). Type 2 diabetes mellitus increases the risk of late-onset Alzheimer's disease: ultrastructural remodeling of the neurovascular unit and diabetic gliopathy. Brain Sci. 9:262. doi: 10.3390/brainsci9100262, 31569571 PMC6826500

[ref24] Hayden M. R. (2024). Cerebral microbleeds associate with brain endothelial cell activation-dysfunction and blood-brain barrier dysfunction/disruption with increased risk of hemorrhagic and ischemic stroke. Biomedicine 12:1463. doi: 10.3390/biomedicines12071463, 39062035 PMC11274519

[ref25] He H. Wang L. Qiao Y. Zhou Q. Li H. Chen S. . (2019). Doxorubicin induces Endotheliotoxicity and mitochondrial dysfunction via ROS/eNOS/NO pathway. Front. Pharmacol. 10:1531. doi: 10.3389/fphar.2019.01531, 31998130 PMC6965327

[ref26] Hernandez-Resendiz S. Palma-Flores C. De Los Santos S. Roman-Anguiano N. G. Flores M. de la Pena A. . (2015). Reduction of no-reflow and reperfusion injury with the synthetic 17beta-aminoestrogen compound Prolame is associated with PI3K/Akt/eNOS signaling cascade. Basic Res. Cardiol. 110:1. doi: 10.1007/s00395-015-0464-y, 25589055

[ref27] Iliff J. J. Wang M. Liao Y. Plogg B. A. Peng W. Gundersen G. A. . (2012). A paravascular pathway facilitates CSF flow through the brain parenchyma and the clearance of interstitial solutes, including amyloid beta. Sci. Transl. Med. 4:147ra111. doi: 10.1126/scitranslmed.3003748 PMC3551275 22896675

[ref28] Infante-Garcia C. Garcia-Alloza M. (2019). Review of the effect of natural compounds and extracts on neurodegeneration in animal models of diabetes mellitus. Int. J. Mol. Sci. 20:2533. doi: 10.3390/ijms20102533, 31126031 PMC6566911

[ref29] Jackson L. Dumanli S. Johnson M. H. Fagan S. C. Ergul A. (2020). Microglia knockdown reduces inflammation and preserves cognition in diabetic animals after experimental stroke. J. Neuroinflammation 17:137. doi: 10.1186/s12974-020-01815-3, 32345303 PMC7189436

[ref30] Ji X. Tian L. Niu S. Yao S. Qu C. (2022). Trimethylamine N-oxide promotes demyelination in spontaneous hypertension rats through enhancing pyroptosis of oligodendrocytes. Front. Aging Neurosci. 14:963876. doi: 10.3389/fnagi.2022.963876, 36072486 PMC9441869

[ref31] Jia R. Sole-Guardia G. Kiliaan A. J. (2024). Blood-brain barrier pathology in cerebral small vessel disease. Neural Regen. Res. 19, 1233–1240. doi: 10.4103/1673-5374.385864, 37905869 PMC11467932

[ref32] Katakam P. V. Snipes J. A. Steed M. M. Busija D. W. (2012). Insulin-induced generation of reactive oxygen species and uncoupling of nitric oxide synthase underlie the cerebrovascular insulin resistance in obese rats. J. Cereb. Blood Flow Metab. 32, 792–804. doi: 10.1038/jcbfm.2011.181, 22234336 PMC3345912

[ref33] Katsi V. Mavroudis A. Liatakis I. Konstantinos M. Tsioufis K. (2024). Exploring the relationship between hypertension and cerebral microvascular disease. Diseases 12:266. doi: 10.3390/diseases12110266, 39589940 PMC11592893

[ref34] Katsumata T. Otori T. Nishiyama Y. Okubo S. Nishiyama Y. Nagayama H. . (2010). Correlation between insulin resistance and white matter lesions among non-diabetic patients with ischemic stroke. Neurol. Res. 32, 743–747. doi: 10.1179/016164109X12608733393755, 20223079

[ref35] Kernan W. N. Viscoli C. M. Furie K. L. Young L. H. Inzucchi S. E. Gorman M. . (2016). Pioglitazone after ischemic stroke or transient ischemic attack. N. Engl. J. Med. 374, 1321–1331. doi: 10.1056/NEJMoa1506930, 26886418 PMC4887756

[ref36] Kim J. K. (2009). Hyperinsulinemic-euglycemic clamp to assess insulin sensitivity *in vivo*. Methods Mol. Biol. 560, 221–238. doi: 10.1007/978-1-59745-448-3_15, 19504253

[ref37] Kosmas C. E. Sourlas A. Oikonomakis K. Zoumi E.-A. Papadimitriou A. Kostara C. E. (2024). Biomarkers of insulin sensitivity/resistance. J. Int. Med. Res. 52:03000605241285550. doi: 10.1177/03000605241285550

[ref38] Kuo P. Y. Tsai H. H. Lee B. C. Chiang P. T. Liu C. J. Chen Y. F. . (2024). Differences in lobar microbleed topography in cerebral amyloid angiopathy and hypertensive arteriopathy. Sci. Rep. 14:3774. doi: 10.1038/s41598-024-54243-1, 38355951 PMC10866968

[ref39] Lee D. H. Lee E. C. Park S. W. Lee J. Y. Lee M. R. Oh J. S. (2024). Pathogenesis of cerebral small vessel disease: role of the Glymphatic system dysfunction. Int. J. Mol. Sci. 25:8752. doi: 10.3390/ijms25168752, 39201439 PMC11354389

[ref40] Lee S. H. Park S. Y. Choi C. S. (2022). Insulin resistance: from mechanisms to therapeutic strategies. Diabetes Metab. J. 46, 15–37. doi: 10.4093/dmj.2021.0280, 34965646 PMC8831809

[ref41] Li D. Li Y. Wang T. Zhu X. (2023). Correlation between insulin resistance and cerebral microbleeds among Chinese patients with cerebral small vessel disease. J. Clin. Neurosci. 111, 1–5. doi: 10.1016/j.jocn.2023.02.018, 37032584

[ref42] Masamoto K. Unekawa M. Watanabe T. Toriumi H. Takuwa H. Kawaguchi H. . (2015). Unveiling astrocytic control of cerebral blood flow with optogenetics. Sci. Rep. 5:11455. doi: 10.1038/srep11455, 26076820 PMC4468581

[ref43] Meng F. Fu J. Zhang L. Guo M. Zhuang P. Yin Q. . (2023). Function and therapeutic value of astrocytes in diabetic cognitive impairment. Neurochem. Int. 169:105591. doi: 10.1016/j.neuint.2023.105591, 37543309

[ref44] Menon S. N. Zerin F. Ezewudo E. Simon N. P. Menon S. N. Daniel M. L. . (2023). Neflamapimod inhibits endothelial cell activation, adhesion molecule expression, leukocyte attachment and vascular inflammation by inhibiting p38 MAPKalpha and NF-kappaB signaling. Biochem. Pharmacol. 214:115683. doi: 10.1016/j.bcp.2023.115683 37429422

[ref45] Nam K. W. Kwon H. M. Jeong H. Y. Park J. H. Kwon H. Jeong S. M. (2020). High triglyceride-glucose index is associated with subclinical cerebral small vessel disease in a healthy population: a cross-sectional study. Cardiovasc. Diabetol. 19:53. doi: 10.1186/s12933-020-01031-6, 32375783 PMC7201807

[ref46] Ngandu T. Lehtisalo J. Solomon A. Levalahti E. Ahtiluoto S. Antikainen R. . (2015). A 2 year multidomain intervention of diet, exercise, cognitive training, and vascular risk monitoring versus control to prevent cognitive decline in at-risk elderly people (FINGER): a randomised controlled trial. Lancet 385, 2255–2263. doi: 10.1016/S0140-6736(15)60461-5, 25771249

[ref47] Ostergaard L. Engedal T. S. Moreton F. Hansen M. B. Wardlaw J. M. Dalkara T. . (2016). Cerebral small vessel disease: capillary pathways to stroke and cognitive decline. J. Cereb. Blood Flow Metab. 36, 302–325. doi: 10.1177/0271678X15606723, 26661176 PMC4759673

[ref48] Ozkan E. Cetin-Tas Y. Sekerdag E. Yigit B. Shomalizadeh N. Sapanci S. . (2023). Hyperglycemia with or without insulin resistance triggers different structural changes in brain microcirculation and perivascular matrix. Metab. Brain Dis. 38, 307–321. doi: 10.1007/s11011-022-01100-7 36305999

[ref49] Pani J. Eikenes L. Reitlo L. S. Stensvold D. Wisloff U. Haberg A. K. (2022). Effects of a 5-year exercise intervention on white matter microstructural Organization in Older Adults. A generation 100 substudy. Front. Aging Neurosci. 14:859383. doi: 10.3389/fnagi.2022.859383, 35847676 PMC9278017

[ref50] Quinones M. J. Nicholas S. B. Lyon C. J. (2005). Insulin resistance and the endothelium. Curr. Diab. Rep. 5, 246–253. doi: 10.1007/s11892-005-0018-z, 16033673

[ref51] Reardon C. A. Lingaraju A. Schoenfelt K. Q. Zhou G. Cui C. Jacobs-El H. . (2018). Obesity and insulin resistance promote atherosclerosis through an IFNγ-regulated macrophage protein network. Cell Rep. 23, 3021–3030. doi: 10.1016/j.celrep.2018.05.010, 29874587 PMC6082182

[ref52] Samuelsson J. Marseglia A. Lindberg O. Westman E. Pereira J. B. Shams S. . (2023). Associations between dietary patterns and dementia-related neuroimaging markers. Alzheimers Dement. 19, 4629–4640. doi: 10.1002/alz.13048, 36960849

[ref53] Sinha S. Haque M. (2022). Insulin resistance is cheerfully hitched with hypertension. Life (Basel) 12:564. doi: 10.3390/life12040564, 35455055 PMC9028820

[ref54] Skrha J. Haas T. Sindelka G. Prazny M. Widimsky J. Cibula D. . (2004). Comparison of the insulin action parameters from hyperinsulinemic clamps with homeostasis model assessment and QUICKI indexes in subjects with different endocrine disorders. J. Clin. Endocrinol. Metab. 89, 135–141. doi: 10.1210/jc.2002-030024, 14715840

[ref55] Son D. H. Lee H. S. Lee Y. J. Lee J. H. Han J. H. (2022). Comparison of triglyceride-glucose index and HOMA-IR for predicting prevalence and incidence of metabolic syndrome. Nutr. Metab. Cardiovasc. Dis. 32, 596–604. doi: 10.1016/j.numecd.2021.11.017, 35090800

[ref56] Starr J. M. Wardlaw J. Ferguson K. MacLullich A. Deary I. J. Marshall I. (2003). Increased blood-brain barrier permeability in type II diabetes demonstrated by gadolinium magnetic resonance imaging. J. Neurol. Neurosurg. Psychiatry 74, 70–76. doi: 10.1136/jnnp.74.1.70, 12486269 PMC1738177

[ref57] Stefanou M. I. Theodorou A. Malhotra K. Aguiar de Sousa D. Katan M. Palaiodimou L. . (2024). Risk of major adverse cardiovascular events and stroke associated with treatment with GLP-1 or the dual GIP/GLP-1 receptor agonist tirzepatide for type 2 diabetes: a systematic review and meta-analysis. Eur. Stroke J. 9, 530–539. doi: 10.1177/23969873241234238, 38400569 PMC11418422

[ref58] Su C. Yang X. Wei S. Zhao R. (2022). Association of Cerebral Small Vessel Disease with Gait and Balance Disorders. Front. Aging Neurosci. 14:834496. doi: 10.3389/fnagi.2022.834496, 35875801 PMC9305071

[ref59] Sun M. Mi W. (2025). Microglial insulin resistance drives neurodegeneration. Trends Endocrinol. Metab. 36, 696–698. doi: 10.1016/j.tem.2025.06.006, 40610268

[ref60] Suren Garg S. Kushwaha K. Dubey R. Gupta J. (2023). Association between obesity, inflammation and insulin resistance: insights into signaling pathways and therapeutic interventions. Diabetes Res. Clin. Pract. 200:110691. doi: 10.1016/j.diabres.2023.110691, 37150407

[ref61] Tanaka M. (2020). Improving obesity and blood pressure. Hypertens. Res. 43, 79–89. doi: 10.1038/s41440-019-0348-x, 31649313

[ref62] Teng Z. Feng J. Dong Y. Xu J. Jiang X. Chen H. . (2022). Triglyceride glucose index is associated with cerebral small vessel disease burden and cognitive impairment in elderly patients with type 2 diabetes mellitus. Front Endocrinol (Lausanne) 13:970122. doi: 10.3389/fendo.2022.970122, 35992100 PMC9390881

[ref63] Teng Z. Feng J. Qi Q. Dong Y. Xiao Y. Xie X. . (2021). Long-term use of metformin is associated with reduced risk of cognitive impairment with alleviation of cerebral small vessel disease burden in patients with type 2 diabetes. Front. Aging Neurosci. 13:773797. doi: 10.3389/fnagi.2021.773797, 34776938 PMC8589019

[ref64] Tian X. Chen S. Wang P. Xu Q. Zhang Y. Luo Y. . (2022). Insulin resistance mediates obesity-related risk of cardiovascular disease: a prospective cohort study. Cardiovasc. Diabetol. 21:289. doi: 10.1186/s12933-022-01729-9, 36564775 PMC9789633

[ref65] van Agtmaal M. J. M. Houben A. de Wit V. Henry R. M. A. Schaper N. C. Dagnelie P. C. . (2018). Prediabetes is associated with structural brain abnormalities: the Maastricht study. Diabetes Care 41, 2535–2543. doi: 10.2337/dc18-1132 30327356

[ref66] Wan S. Dandu C. Han G. Guo Y. Ding Y. Song H. . (2023). Plasma inflammatory biomarkers in cerebral small vessel disease: a review. CNS Neurosci. Ther. 29, 498–515. doi: 10.1111/cns.14047, 36478511 PMC9873530

[ref67] Wang X. Li R. Zacharek A. Landschoot-Ward J. Chopp M. Chen J. . (2019). ApoA-I mimetic peptide reduces vascular and white matter damage after stroke in Type-2 diabetic mice. Front. Neurosci. 13:1127. doi: 10.3389/fnins.2019.01127, 31708728 PMC6823666

[ref68] Wardlaw J. M. Smith E. E. Biessels G. J. Cordonnier C. Fazekas F. Frayne R. . (2013). Neuroimaging standards for research into small vessel disease and its contribution to ageing and neurodegeneration. Lancet Neurol. 12, 822–838. doi: 10.1016/S1474-4422(13)70124-8, 23867200 PMC3714437

[ref69] Wardlaw J. M. Smith C. Dichgans M. (2019). Small vessel disease: mechanisms and clinical implications. Lancet Neurol. 18, 684–696. doi: 10.1016/S1474-4422(19)30079-1, 31097385

[ref70] Wu T. D. Fawzy A. Brigham E. McCormack M. C. Rosas I. Villareal D. T. . (2021). Association of triglyceride-glucose index and lung health: a population-based study. Chest 160, 1026–1034. doi: 10.1016/j.chest.2021.03.056, 33839084 PMC8449007

[ref71] Wu D. Yang X. Zhong P. Ye X. Li C. Liu X. (2020). Insulin resistance is independently associated with enlarged perivascular space in the basal ganglia in nondiabetic healthy elderly population. Am. J. Alzheimers Dis. Other Dement. 35:1533317520912126. doi: 10.1177/1533317520912126, 32180437 PMC10624068

[ref72] Wu L. Zhu J. Li C. Zhu J. Dai Z. Jiang Y. (2022). Association of triglyceride-glucose index with ischemic stroke recurrence in nondiabetic patients with small vessel occlusion: a multicenter hospital-based prospective cohort study. Cardiovasc. Diabetol. 21:250. doi: 10.1186/s12933-022-01693-4, 36397084 PMC9673408

[ref73] Yang X. Zhang S. Dong Z. Zi Y. Luo Y. Jin Z. . (2019). Insulin resistance is a risk factor for overall cerebral small vessel disease burden in old nondiabetic healthy adult population. Front. Aging Neurosci. 11:127. doi: 10.3389/fnagi.2019.00127, 31249520 PMC6582254

[ref74] Zhang D. D. Cao Y. Mu J. Y. Liu Y. M. Gao F. Han F. . (2022). Inflammatory biomarkers and cerebral small vessel disease: a community-based cohort study. Stroke Vasc Neurol 7, 302–309. doi: 10.1136/svn-2021-001102, 35260438 PMC9453831

[ref75] Zhang C. E. Wong S. M. Uiterwijk R. Backes W. H. Jansen J. F. A. Jeukens C. . (2019). Blood-brain barrier leakage in relation to white matter hyperintensity volume and cognition in small vessel disease and normal aging. Brain Imaging Behav. 13, 389–395. doi: 10.1007/s11682-018-9855-7, 29572621 PMC6486901

[ref76] Zhou M. Mei L. Jing J. Yang Y. Cai X. Meng X. . (2024). Blood pressure partially mediated the Association of Insulin Resistance and Cerebral Small Vessel Disease: a community-based study. J. Am. Heart Assoc. 13:e031723. doi: 10.1161/JAHA.123.031723, 38390815 PMC10944068

[ref77] Zhou M. Wang S. Jing J. Yang Y. Cai X. Meng X. . (2022). Insulin resistance based on postglucose load measure is associated with prevalence and burden of cerebral small vessel disease. BMJ Open Diabetes Res. Care 10:e002897. doi: 10.1136/bmjdrc-2022-002897, 36220196 PMC9557259

[ref78] Zhu H. H. Wang Y. C. He L. C. Luo H. Y. Zong C. Yang Y. H. . (2024). Novel inflammatory and insulin resistance indices provide a clue in cerebral amyloid angiopathy. Sci. Rep. 14:11474. doi: 10.1038/s41598-024-62280-z, 38769356 PMC11106308

